# A Human Development Framework for CO_2_ Reductions

**DOI:** 10.1371/journal.pone.0029262

**Published:** 2011-12-21

**Authors:** Luís Costa, Diego Rybski, Jürgen P. Kropp

**Affiliations:** Potsdam Institute for Climate Impact Research, Potsdam, Germany; University of Oxford, United Kingdom

## Abstract

Although developing countries are called to participate in CO_2_ emission reduction efforts to avoid dangerous climate change, the implications of proposed reduction schemes in human development standards of developing countries remain a matter of debate. We show the existence of a positive and time-dependent correlation between the Human Development Index (HDI) and per capita CO_2_ emissions from fossil fuel combustion. Employing this empirical relation, extrapolating the HDI, and using three population scenarios, the cumulative CO_2_ emissions necessary for developing countries to achieve particular HDI thresholds are assessed following a Development As Usual approach (DAU). If current demographic and development trends are maintained, we estimate that by 2050 around 85% of the world’s population will live in countries with high HDI (above 0.8). In particular, 300 Gt of cumulative CO_2_ emissions between 2000 and 2050 are estimated to be necessary for the development of 104 developing countries in the year 2000. This value represents between 20 % to 30 % of previously calculated CO_2_ budgets limiting global warming to 2°C. These constraints and results are incorporated into a CO_2_ reduction framework involving four domains of climate action for individual countries. The framework reserves a fair emission path for developing countries to proceed with their development by indexing country-dependent reduction rates proportional to the HDI in order to preserve the 2°C target after a particular development threshold is reached. For example, in each time step of five years, countries with an HDI of 0.85 would need to reduce their per capita emissions by approx. 17% and countries with an HDI of 0.9 by 33 %. Under this approach, global cumulative emissions by 2050 are estimated to range from 850 up to 1100 Gt of CO_2_. These values are within the uncertainty range of emissions to limit global temperatures to 2°C.

## Introduction

Consensus emerging in favor of low CO_2_ stabilization targets requires the participation of developing countries in the efforts to reduce global green-house emissions [1]. For example, it has been claimed that in order to keep global temperatures below a 2°C increase, developing countries should attain more than 20 % CO_2_ reductions below business-as-usual levels by the year 2020 [2]. The potential implications of such reductions on development standards remain unclear [3] as developing countries are expected to extensively rely on fossil energy to fuel their current development needs [4]. In addition to potential development implications, a fair allocation of responsibility regarding CO_2_ emissions reduction between developed and developing countries remains a controversial topic [5], [6]. How to account for the responsibility of developed countries regarding historical CO_2_ emissions [7] and to what extent technological and political inertia impose limits to the range of strategies envisioning the implementation of reduction schemes [8] are questions that remain largely unanswered. Developing countries have expressed their concerns on the points raised, questioning if development goals can – or cannot – be met under current technological and population trends [9].

In order to tackle above mentioned challenges, the CO_2_ allocation and reduction approach here outlined contrasts from existing ones [5], [7], [10] by relying on the Human Development Index (HDI) [11] as a summary measure reflecting the achievement of a country in three basic dimensions of human development: a long healthy life, access to knowledge, and decent living standards. These dimensions are assessed based on the following indicators: life expectancy at birth, literacy rate of adults, gross enrollment rate, and gross domestic product per capita at purchasing power parity [11]. Despite some criticism – for example treating income, health, and education as substitutes [12] – the HDI has been consistently used by the United Nations Development Programme (UNDP) as a reference metric to compare social and economic development within and between countries across time. Furthermore, the HDI has been reported to play an important role in raising the political profile of general health and educational policies [13], to be an indicator of a country’s exposure to climate-related extremes [14] and its dimensions determinants of vulnerability and adaptive capacity at national level [15].

**Figure 1:**
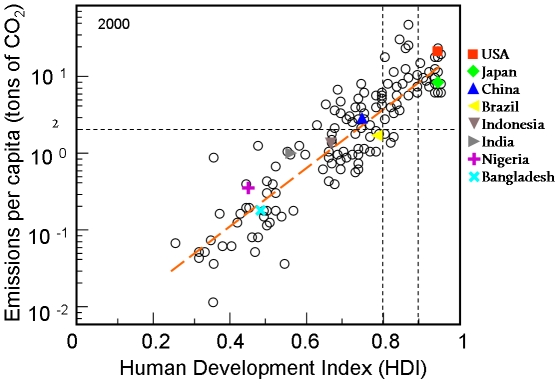
Correlations between HDI and CO_2_ per capita emissions in the year 2000. The dashed line represents a least squares fit through all values. The coefficient of determination is 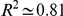 and the correlation coefficient is 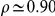. For some countries the values are shown explicitly. Vertical lines represent the HDI values of 0.8 and 0.9 representative of high and very high development standards respectively as expressed in the United Nations Development Report 2009 [34]. The horizontal line shows the 2 tons per capita CO_2_ emissions target to limit global warming at 2°C by 2050 [7].

In Figure 1 per capita emissions are plotted against the corresponding HDI for countries with available data in the year 2000. We find that the per capita CO_2_ emissions from fossil fuel burning are exponentially correlated with human development – highlighting the often disregarded social-dimension of emissions reductions. For example, the development strategy targeting high growth in domestic product by relying on low-cost, low-efficiency technology, contributed for the poverty rate in China to drop from 53% in 1981 to 8% in 2001 [16]. Although this “fossil” path of development is highly incompatible with future climate targets, climate policies cannot neglect the potential societal implications of CO_2_ reductions, especially during the first stages of human development in a country. The magnitude of the challenges ahead become clear once the per capita CO_2_ emissions guard rail of 2 tons for avoiding dangerous climate change [17] and the HDI thresholds of 0.8 and 0.9 (characteristic of a developed world) are displayed. A fair distribution of CO_2_ emissions under current technological constraints should allow the convergence of developing countries towards 0.8 or 0.9 HDI scores and, at the same time, keep global CO_2_ emissions below the available budgets limiting anthropogenic climate change.

## Methods

### Extrapolating the Human Development Index

**Figure 2:**
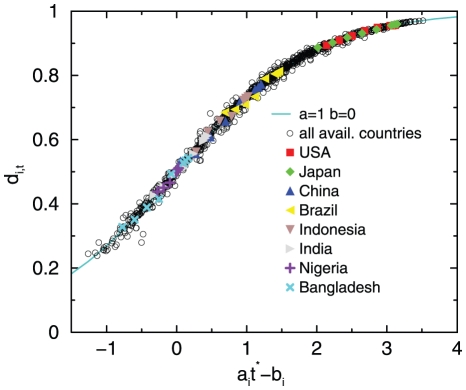
Collapse of the HDI values based on logistic regression according to Eq. (1). HDI values are plotted for each country by using a transformed time 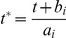 so that HDI values of all countries (open circles) fall within their spreading on the curve which is used to fit the data. The filled symbols intend to highlight the same examples as in Fig. 1. The solid line corresponds to the function 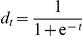.

Our approach starts by investigating the evolution of future human development standards. We assume that the HDI, *d_i,t_*, of a country, *i*, evolves in time, *t*, following a logistic regression [18]. This choice is supported by the fact that the HDI is bounded to 0≤*d_i,t_*≤1 and that countries with high HDI evolve slowly in time. Further, this asymptotic behavior suggests the existence of smooth transitions in development. The logistic regression fulfills these requirements. Therefore, we fit for each country separately 
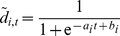
(1)to the available data (obtaining the parameters *a_i_* and *b_i_*). Since the regression involve only two parameters, three measures of HDI would suffice to over-determine the system. We have chosen to use countries for which we have at least four values of HDI in order to obtain more robust results. This lead to regressions for 147 countries out of 173 in our data set. Basically, *a_i_* quantifies how fast a country develops and *b_i_* represents a delay. In Figure 2 we display the collapse (see e.g. [19]) of the past HDI as obtained from the logistic regressions illustrating how countries have been developing in the scope of this approach. The HDI values of each country are plotted using a transformed time 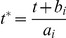 so that values of all countries (open circles) fall within their spreading on the curve which is used to fit the data. The filled symbols highlight the same countries as in Figure 1. The solid line corresponds to the function 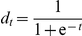. Based on the obtained parameters, *a_i_* and *b_i_*, we estimate the future HDI of each country until 2050 assuming similar development trajectories as in the past.

### Projecting per capita emissions

In the following section we provide the main assumptions used to extrapolate per capita emissions of CO_2_ from fossil fuel burning (see also section III.B in Text S1). We choose not to include emissions from other greenhouse gases since they were found not to be strongly correlated with personal consumption and national carbon intensities [6]. CO_2_ emissions from land-use were disregarded due to the high uncertainty of historical data [20].

The correlations between HDI and CO_2_ emissions per capita, 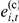, were assessed for all years (1980-2006), see example of Figure 1. We apply the exponential regression 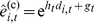
(2)to the data by linear regression [21] through 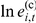 versus *d_i,t_* for fixed years *t* and obtain the parameters *h_t_* and *g_t_*. At a global level, correlation coefficients varied between a minimum of 0.89 in 2005 and a maximum of 0.91 in 2006. The individual components of HDI were found to be as well correlated with per capita emissions, in the following decreasing order of correlation coefficient: GDP, education, and life expectancy, see Figure S2 and Table S2 in Text S1.

We take advantage of these correlations and assume that the system is ergodic, i.e. that the process over time and over the statistical ensemble is the same. In other words, we assume that these correlations also hold for each country individually and apply the exponential regression 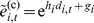
(3)obtaining the parameters *h_i_* and *g_i_*, which are now country dependent. Based on the estimated parameters the CO_2_ per capita emissions are extrapolated country wise. We additionally tested two population-weighing methods when fitting per capita emissions versus the HDI (see section III.E and Figure S7 in the Text S1).

For 52 countries out of 173 data was found to be insufficient to perform the regressions Eq. (1) or Eq. (3). This is, they comprise less than the minimum number of data points required to fit the HDI versus time or CO_2_ emissions per capita versus HDI. In the Text S1 (see section III.C and Figures S3 and S4), we find that changes of *d_i,t_* and 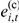 are correlated among the countries. Thus, in the 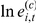
- *d_i,t_*-plane, we let countries with a lack of data evolve in a similar way as those in their vicinity.

**Figure 3:**
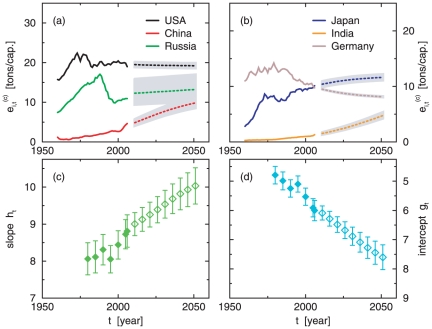
Examples of extrapolated CO_2_ per capita emissions. Panels **a** and **b** show the extrapolated values of CO_2_ emissions per capita for 6 countries following a DAU approach. The gray uncertainty range is obtained by including the statistical errors of the regressions (one SD each). Panels **c** and **d** represent the slope and intercept values for the ensemble regressions of HDI versus CO_2_ per capita for observed (filled symbols) and projected (open symbols) data. The error bars are given by the standard errors.

In Figure 3 the panels (a) and (b) show examples of extrapolated CO_2_ emissions per capita for six countries according to the described methodology (more examples can be found in Figures S1 and S6 of the Text S1). Measured values (solid lines) and extrapolated values are plotted up to the middle of the 21st century (dashed lines). The gray uncertainty range is obtained by including the statistical errors of the regressions (one Standard Deviation (SD) each). For the set of countries for which data is available we obtain the parameters *h_t_* and *g_t_* as displayed in the panels (c) and (d) of Figure 3 for the past values (filled symbols) and for projected values (open symbols). The parameters imply that in average, for a given HDI, the corresponding CO_2_ emissions decrease during the time frame under investigation, as can also be seen in Figure S5 of the Text S1. It is apparent that these correlations are hard to overcome since they are intrinsic to the energy supply systems.

Future country-based emissions estimates are obtained via multiplying the extrapolated CO_2_ per capita values by population numbers extracted from three scenarios published in the Millennium Ecosystem Assessment report [22]. For the purpose of this work we only make use of data until 2050 and the population scenarios Adaptive Mosaic (AM), Technogarden (TG), and Global Orchestration (GO).

The statistical approach undertaken in this work can be named “Development As Usual” (DAU) in the sense that development and emission trends continue as in the past. Accordingly, we are not claiming that the calculated HDI and CO_2_ extrapolations are predictions, instead, they represent a plausible near-future world (by 2050) where CO_2_ emissions from fossil fuel combustion are still closely linked to human development. This assumption is supported by (i) the findings that no discernible decarbonizing trends of energy supply among world regions can be identified [23] and (ii) the existence of substantial obstacles to large scale implementation of renewable energy in the near future [24].

## Results

### Emissions for development

**Figure 4:**
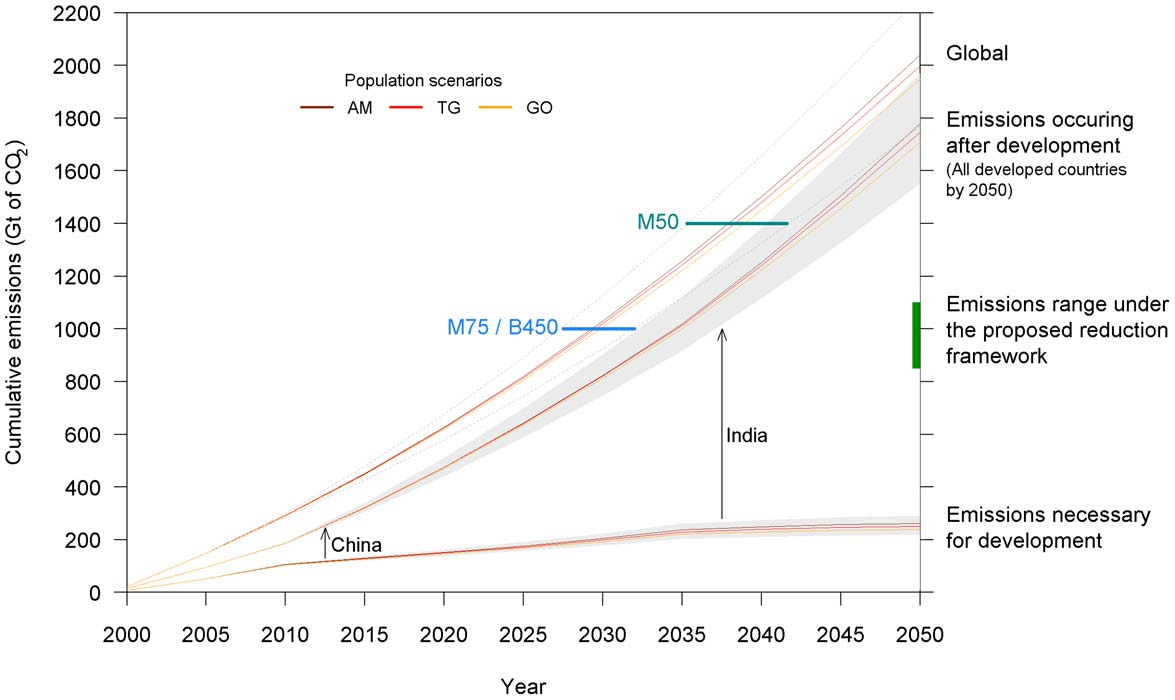
Cumulative CO_2_ emissions for Development As Usual (DAU) according to three population scenarios. Global emissions are split into two emission budgets: emissions necessary for development (until an HDI of 0.8 is reached) and emissions occurring after development (all developed countries in 2050). Population scenarios are extracted from the Millennium Ecosystem Assessment report [22] (AM – Adaptive Mosaic; TG – Technogarden; GO – Global Orchestration). Horizontal lines illustrate the representative values of cumulative CO_2_ emissions associated with the probabilities 75% and 50% (M75 and M50) of staying below the 2°C target by 2050 as provided by Meinshausen *et al.* [25] and cumulative emission budgets required to stabilize CO_2_ concentrations at 450 ppm provided by Broecker [5] (B450). The black arrows represent two illustrative examples (China and India) and indicate the estimated time frame when the HDI threshold of 0.8 is crossed and emissions are no longer accounted to be necessary for development. The green bar at the right edge of the frame depicts the range of cumulative emissions achievable under the proposed reduction framework.

Figure 4 depicts the estimated cumulative emissions for the three population scenarios together with a set of CO_2_ budgets for particular warming and concentration targets [5], [25], [26]. According to the DAU approach, global cumulative CO_2_ emissions by 2050 range from 1700 up to 2300 Gt of CO_2_ with about 85% of the world’s population living in countries with an HDI above 0.8. When assessed on a per year basis, emissions range between 45.6 and 62.4 Gt CO_2_ in 2050 (corresponding respectively to 12.5 and 17.1 Gt of carbon in 2050, using factor 44/12 for conversion [27]), which is within the range of recent projections using IPCC emissions scenarios [28], [29].

Of a total of 165 countries, 104 were found to be developing countries (HDI below 0.8) in the year 2000. By using the UNDP HDI threshold of 0.8 to differentiate countries with high human development from developing countries with medium to low human development [30], estimated global CO_2_ emissions are divided into two budgets. The first budget includes the emissions necessary for the development of countries with HDI below 0.8 while the second budget accounts for emissions occurring after development, this is, emissions from countries with HDI above 0.8. Emissions from countries carrying out a development transition (i.e., crossing the HDI threshold between 2000 and 2050) are added correspondingly to each budget. For example, we estimate India to achieve an HDI above 0.8 between the years 2035 and 2040 (see Table S1 for the time periods when countries undertake a development transition). Until the HDI threshold is reached the emissions are accounted to be necessary for development, from then on CO_2_ emissions from India are accounted to occur after development.

In a DAU future we estimate that between 200 and 300 Gt of cumulative CO_2_ emissions will be necessary for developing countries (104 in the year 2000) to proceed with their development. In the scope of our approach, 61 developing countries are expected to overcome the HDI of 0.8 by 2050 consuming roughly 98 % of the above-mentioned 200-300 Gt budget. The remaining 43 countries are likely to stay below the UNDP high human development threshold in the considered time frame. Total cumulative emissions occurring after development range from 1500 to 2000 Gt of CO_2_.

This amount is similarly divided among countries carrying out a development transition (700 to 1000 Gt) and those whose development occurred before the year 2000 (800 to 1000 Gt) as summarized in Table 1.

**Table 1:** Projected cumulative CO_2_ emissions for the period 2000-2050 compared to CO_2_ emission budgets for warming potential and atmospheric concentrations.

	Cumulative CO_2_ emissionsin Gt of CO_2_ by 2050		
Necessary for development*	**200**	**-**	**300**
Emitted after development	**1500**	**-**	**2000**
from countries crossing 0.8 HDI between 2000 and 2050	700	-	1000
from countries already developed in 2000	800	-	1000
Global			
Emissions under DAU	**1700**	-	**2300**
Emissions under the proposed framework**	**850**	-	**1100**

	Allowable CO_2_ emissionsin Gt of CO_2_
By 2050	
75% probability of not exceeding 2°C [25] (M75)	**1000**
50% probability of not exceeding 2°C [25] (M50)	**1400**
By 2075	
To limit CO_2_ concentrations at 450 ppm [5] (B450)	**1000**
To limit CO_2_ concentrations at 560 ppm [5]	**2600**
To limit CO_2_ concentrations at 560 ppm [26]	**3300**

The table summarizes the emission values before and after countries reach the HDI of 0.8 according to a DAU approach and under the proposed reduction framework. A collection of previous calculated budgets for allowable CO_2_ emissions highlights the efforts necessary for emission reductions.

*Cumulative emissions necessary for development assuming an HDI threshold of 0.9 would range from 600 to 600 Gt CO_2_.

**Assuming the same uncertainty as in DAU.

Emissions for development where found to be very sensitive to the selected HDI score. Assuming that developing countries want to achieve western development styles would require to set the minimum development standards to values of 0.9. In such a case, emissions necessary for development by 2050 range from about 700 to 900 Gt of CO_2_. This range is higher by at least a factor of 3 than the values obtained for a HDI threshold of 0.8.

We further compare our estimates with previously calculated CO_2_ budgets for particular time frames, global warming targets and atmospheric CO_2_ concentrations. We find that the emissions necessary for development consume up to 30 % of the 1000 Gt CO_2_ limit for a 75 % probability of keeping global warming below 2°C, as calculated by Meinshausen *et al.* [25] and indicated as M75 in Fig. 4. According to our projections, the 1000 Gt budget limit by 2050 would already be exhausted around 2030 if human development proceeds as in the past. In case one adopts the CO_2_ limit providing a 50 % chance (M50) of staying below 2°C, then cumulative CO_2_ emissions necessary for development would still represent about 20 % of the total budget. Similarly, the CO_2_ budget to stabilize atmospheric concentrations at 450 ppm provided by Broecker [5] (indicated as B450 in Fig. 4), would be exhausted within the next 20 years.

### Human development framework for CO_2_ allocation and reduction

**Figure 5:**
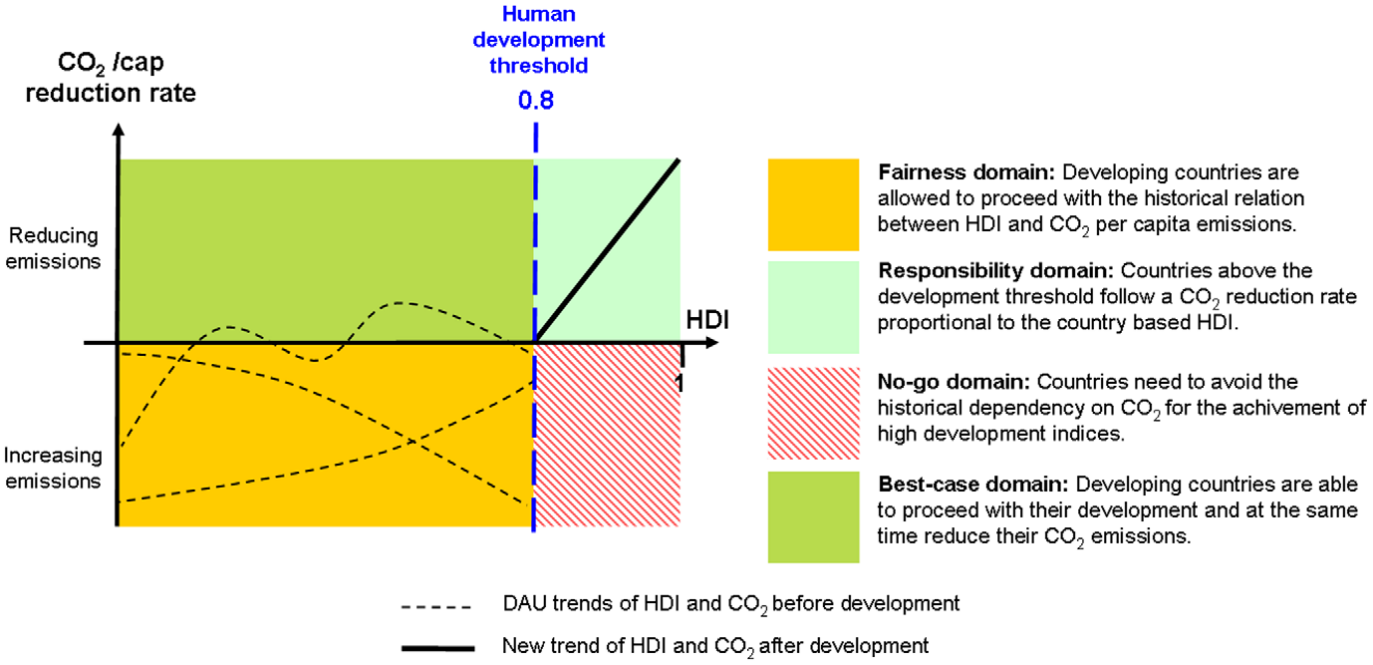
CO_2_ emissions reduction framework based on HDI. The reduction framework proposes four domains of climate action that are both fair in an historical perspective and constrained by current technological developments. Reserving a fairness domain for developing countries implies that their participation in climate efforts can be operated in a voluntary basis. The development threshold of 0.8 HDI is taken from United Nations Development Report 2009 [34].

The question logically arising from the results is how to operate a fair transition of developing countries towards high development standards without compromising current climate targets. A fair approach implies that an hypothetical developing country should not be limited in its emissions of CO_2_ until it reaches a particular threshold of human development. In practice, the development path made by current developed countries in the past should be possible for developing countries in the future if they choose to do so. This key aspect of the proposed framework convenes in our opinion a better representation of fairness in CO_2_ emissions allocation as opposed to fixing a point in the past from where emissions are integrated. Figure 5 makes use of the 0.8 HDI threshold to differentiate four areas of action regarding climate policies. Countries whose HDI trails below the minimum human development standard evolve in the context of a *Fairness domain*. In this domain the developing country is allowed to fulfill the basic development needs by following a development path where HDI is highly correlated with CO_2_ emissions from fossil fuel burning. In the *Best-case domain* developing countries are able to proceed with their development goals and at the same time reduce their CO_2_ emissions. This domain would imply a fast worldwide implementation of energy technologies with low carbon intensity, a transformation that is not observed so far [23]. After basic development needs are fulfilled, countries are no longer said to be developing and transit to the *Responsibility domain* where they engage in CO_2_ reduction rates proportional to their HDI in order to preserve a global warming limit of 2°C by 2050 [25]. The *No-go domain* needs to be avoided by future developed countries and quickly abandoned by current ones on the basis that resulting emissions would be largely incompatible with future climatic policies. A generalized convergence of countries towards the *Responsibility domain* should be operated.

To formalize this, we propose that a developed country *i* reduces it’s per capita emissions at year *t* according to 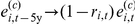 with the 5-year reduction rate *r_i,t_*, given by 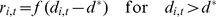
(4)where *d* ^*^ is the development threshold and *f* a proportionality constant which determines how strong the reduction rate increases with increasing HDI (see also Text S1 section V). Based on the above discussed development threshold (*d* ^*^ = 0.8) we estimate that *f*  =  3.3 (as a lower bound) would lead to global cumulative emissions ranging between 850 and 1100 Gt of CO_2_ by 2050 if reduction starts in 2015 (assuming the same uncertainty as in DAU). This amount is within the range of allowed cumulative CO_2_ emissions that provide between 80 % and 66 % change of keeping global temperatures below a 2°C increase, as calculated by [25]. Under our reduction framework, global emissions in the year 2050 are estimated to be 10 Gt CO_2_ or about 13.3 Gt CO_2_ equivalent if one accounts also for non-CO_2_ gases (with non-CO2 gases constituting roughly 1/3 of total CO_2_ equivalent [25]). This value is relatively low and complies with post-2050 emission thresholds that make cumulative CO_2_ emissions between 2010 and 2050 a robust indicator of achieving the 2°C target as in Meinshausen *et al.* [25] and Bowerman *et al.* [27].

**Figure 6:**
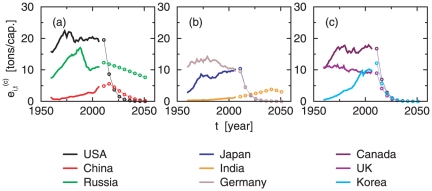
Examples of extrapolated CO_2_ emissions per capita in agreement with the proposed reduction scheme (*d* ^*^ = 0.8, *f* = 3.3). Solid lines stand for the historical emission while the connected circles represent extrapolated emissions when countries follow the reduction scheme proposed.

**Figure 7:**
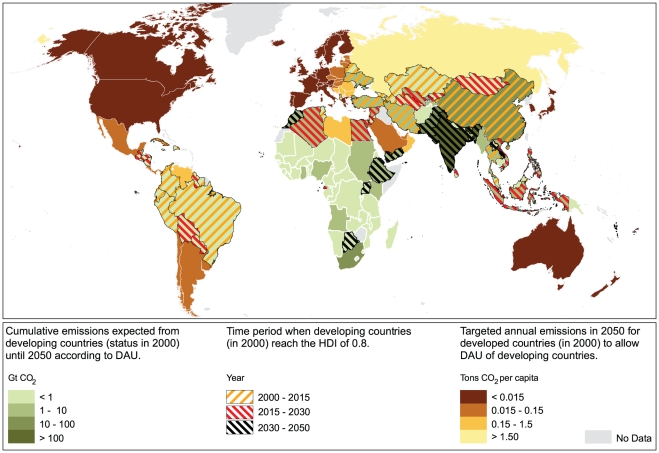
Global distribution of allowed emissions for DAU from developing countries (green shading) and per capita CO_2_ targets in 2050 for developed countries (brown shading) under the proposed framework to keep temperatures below 2°C target – as implied by the M75 CO_2_ budget. The period in time when developing countries are expected to reach an HDI of 0.8 is represented by the colored hatches.

The value of *f*  =  3.3 implies that in each time step of five years, countries with an HDI of 0.85 would need to reduce their per capita emissions by approx. 17% and countries with an HDI of 0.9 by 33 %. As a result of applying these reduction rates, emission curves of current and future developed countries decrease approximately exponentially. In Figure 6 we show the emission trajectories for a set of countries. Per capita CO_2_ emissions from Germany would need to be reduced from about 10 tons in 2010 to 4 tons in 2020 and a nearly complete decarbonization by 2040. Countries not yet developed are entitled to increase emissions. In the case of India, CO_2_ emissions per capita grow until a maximum of 4 tons in 2040. After its development, India needs to reduce per capita emissions to approx. 3.5 and 3 tons CO_2_ in 2045 and 2050 respectively. Developing countries unable to reach an HDI of 0.8 during the time frame of this analysis are allowed to emit following DAU. For example, Pakistan is entitled to increase emissions to a maximum of approx. 2.5 tons per capita in 2050, the year when its expected to become a developed country following our approach. In Figure 7 we provide an overview of our results according to the current political world map. The figure highlights the geographic trade-offs between the necessary achievements in CO_2_ reduction by current developed countries (brown shading), and the cumulative CO_2_ emissions for the DAU of developing countries (green shading) in order to comply with the 2°C target – using the M75 budget.

## Discussion

Previous reduction schemes of global CO_2_ emissions make use of population numbers [5], [7], [31] or income distribution [10] associated with permissible CO_2_ atmospheric concentrations or global warming targets [32] to comply with the “common but shared responsibility” principle of the 1992 United Nations Framework Convention on Climate Change (UNFCCC).

These approaches disregard to some extent the possible development set-backs caused by CO_2_ reductions in the socio-economic development of a country. We use the HDI in order to take development needs of developing countries into consideration. In a DAU world, we estimate that up to 300 Gt of CO_2_ represent a pre-condition for raising a considerable amount of developing countries (comprising HDI below 0.8 in the year 2000) to a minimum HDI of 0.8 in the year 2050. If development pathways proceed as in the past, resulting CO_2_ emissions will pose tighter constraints on the achievement of the previously mentioned climate targets. One can legitimately question the likelihood of such assumption. In a sense, our approach can only be regarded as an approximation since aspects like technological innovation and enhanced technology transfer between developed and developing countries cannot be anticipated. This is a recurrent problem when projecting trends of socio-economic systems into the future. The assumed ergodicity would benefit from further investigation.

Depending on mankind’s decision concerning acceptable levels of climate change and desirable human development goals, emissions necessary for development can represent substantial shares of the CO_2_ budgets here analyzed (see Table 1 for further CO_2_ budgets). In line with previous research [33], it was found that the overall efficiency in achieving higher human development scores increases, e.g. less CO_2_ emissions are necessary for a certain HDI. It remains open to which extent these gains in efficiency can be articulated in the context of current climate negotiations constraints.

We propose a differentiated and dynamic allocation scheme of CO_2_ emissions based on human development achievements. Developing countries are not obliged to reduce their emissions until a certain threshold of human development is achieved. From then on the country is no longer considered to be developing, and should therefore engage on the proposed emissions reduction path. It is worth to point out that the investigated population scenarios only show substantial divergence in values beyond 2050. Obtained differences in CO_2_ emissions between scenarios are therefore small during the time frame of analysis.

Within the scope of our approach the efforts for climate protection commitments from developing countries can be operated on a voluntary basis. With CO_2_ reduction rates linked to the evolution of HDI as proposed here, the 2°C target can be met even if emissions from developing countries evolve according to DAU during the early stages of development. Independent of the climate target, a fair allocation and reduction of emissions between developed and developing countries must consider the dependence between CO_2_ and human development here discussed.

## Supporting Information

Figure S1 **Examples of extrapolated CO_2_ emissions per capita.** For the countries with top total emissions in 2000, we plot the measured values (solid lines) and extrapolated values up to the middle of the 21st century (dotted lines). The gray uncertainty range is obtained by including the statistical errors of the regressions (one standard deviation each).
(EPS)

Figure S2 **Correlations between CO_2_ emissions per capita and HDI as well as its components.** Panels (a-d) are cross-plots in semi-logarithmic representation, where each filled circle represents a country. (a) depicts the CO_2_ emissions per capita values versus the corresponding HDI values for the year 2006 (172 countries). (b-d) depict the analogous for the HDI components, i.e. (b) GDP index, (c) life expectancy index, and (d) education index. The slopes and correlation coefficients are listed in Table S2. The Panels also include the trajectories (1980-2006) of Japan (green diamonds), China (blue triangle up), India (grey triangle right), and Bangladesh (cyan ×) evolving from the lower left to the upper right. The solid straight lines are exponential fits, Eq. 4), to the data and the dotted lines in (b-d) correspond to the fit from (a).
(EPS)

Figure S3 **Correlations of the changes in development and emissions per capita.** For observed data between the years 2000 and 2005, we plot in (a) the correlation function, Eq. 6), of the temporal changes of the HDI as a function of the difference of the countries in terms of HDI. In (b) the analogue, namely the correlation function of the temporal changes of the emissions per capita is plotted as a function of the difference in terms of emissions per capita. While the green dots represent the products of individual pairs, the blue filled circles represent the averages in logarithmic bins.
(EPS)

Figure S4
**Correlations between CO_2_ emissions per capita and HDI.** Panels (a) to (g) are cross-plots in semi-logarithmic representation, where each filled circle represents a country, for past years (a) 2000:135 countries and (b) 2006: 172 countries, as well as extrapolated (c) to (g) 2011-2051 (172 countries each). The brownish circles represent those countries, which due to missing data have been estimated assuming correlations in the changes of *d_i,t_* as well as 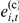 (see Sec. III.C). Panels (h) and (i) show how the parameters *h_t_* and *g_t_* evolve in time (the open symbols are obtained from the extrapolated values of all countries). Both parameters are based on only those 71 countries providing HDI and CO_2_ values for all years 1980, 1985, 1990, 1995, 2000, 2005, 2006. The qualitative agreement of *h_t_* and *g_t_* between past and extrapolated supports the plausibility of the presented approach. The error bars are given by the standard errors. The panels (h) and (i) are the same as Fig. 3(c) and (d) in the main text.(EPS)

Figure S5 **Evolving correlations between CO_2_ emissions per capita and HDI.** The lines represent the linear regressions applied to the logarithm of CO_2_ emissions per capita versus HDI for the past (solid lines) and our projections (dashed lines). The numbers at the right edge correspond to the *e_i,t_* for which the regressions cross *d_i,t_* = 0.8 in 1980, 2005 and projected for 2051.
(EPS)

Figure S6
**Examples of extrapolated CO_2_ emissions per capita.** For the countries with top emissions per capita in 2006, we plot the measured values (solid lines) and extrapolated values up to the middle of the 21st century (dotted lines). Qatar and Luxembourg belong to those countries, which due to missing data have been extrapolated utilizing correlations in the changes of *d_i,t_* as well as 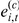 (Sec. III.C). The gray uncertainty range is obtained by including the statistical errors of the regressions (one standard deviation each). Analogous to Figure S1 but for different countries.(EPS)

Figure S7 **Correlations between CO_2_ emissions per capita and HDI.** For the year 2000 three different ways of performing a regressions are exemplified. Solid line in the background: the regression when each country has the same weight. Dotted line: the countries have weights according to the logarithm of their population. Dashed line: the countries have weights according to their population. For comparison the five most populous countries are highlighted.
(EPS)

Table S1 **Periods during which countries are expected to pass the HDI value of 0.8 according to the extrapolations.** The rows denote the countries and the columns denote periods of five years. The transitions are indicated with •.
(PDF)

Table S2 **Slopes and correlation coefficients of the exponential fits, Eq. 4), applied to the HDI and it’s components.**
(PDF)

Text S1 (PDF)
