# Supplementary material for: A Human Development Framework for CO2 Reductions
Source: PLoS One. 2011 Dec 21;6(12):e29262. doi: 10.1371/journal.pone.0029262 (PMC3244443; doi:10.1371/journal.pone.0029262)
Supplement: Table S2 — Slopes and correlation coefficients of the exponential fits, Eq. 4), applied to the HDI and it’s components. (PDF) [file pone.0029262.s009.pdf]

component	slope h	corr. coeff.
HDI	8.93 ± 0.31	0.91
GDP	7.34 ± 0.24	0.92
life exp.	7.86 ± 0.48	0.78
education	7.87 ± 0.41	0.83
