# Supplementary material for: A Human Development Framework for CO2 Reductions
Source: PLoS One. 2011 Dec 21;6(12):e29262. doi: 10.1371/journal.pone.0029262 (PMC3244443; doi:10.1371/journal.pone.0029262)
Supplement: Text S1 — (PDF) [file pone.0029262.s010.pdf]

SUPPORTING INFORMATION (SI)
A Human Development Framework for CO_2 Reductions

Luís Costa,* Diego Rybski,† and Jürgen P. Kropp‡
Potsdam Institute for Climate Impact Research (PIK), P.O. Box 60 12 03, 14412 Potsdam, Germany
(Dated: November 25, 2011)

Contents

I. Synopsis 1

II. Data 1
A. Human Development Index (HDI) 2
B. CO_2 emissions per capita 2
C. Population 2
D. Notation 2

III. Extrapolating CO_2 emissions 2
A. Extrapolating Human Development Index (HDI) 3
B. Estimating CO_2 emissions per capita 3
1. Correlations between CO_2 emissions per capita and HDI components 3
C. Estimating values for missing countries 4
D. Uncertainty 4
E. Limitations 5
F. Enhanced development approach 5

IV. Cumulative emissions 5

V. Reduction scheme 5

Acknowledgments 6

References 6

I. SYNOPSIS

We estimate cumulative CO_2 emissions during the period 2000 to 2050 from developed and developing countries based on the empirical relationship between CO_2 per capita emissions (due to fossil fuel combustion and cement production) and corresponding HDI. We choose not to include emissions from land use and other greenhouse gases since they were found not to be strongly correlated with personal consumption and national carbon intensities [1]. In addition, data of past CO_2 emissions from land use is uncertain due to the lack of historical data of both former ecosystem conditions and the extent of subsequent land use [2].

In order to project per capita emissions of individual countries we make three assumptions which are detailed below. First, we use logistic regressions to fit and extrapolate the HDI on a country level as a function of time. This is mainly motivated by the fact that the HDI is bounded between 0 and 1 and that it decelerates as it approaches 1. Second, we employ for individual countries the correlations between CO_2 per capita emissions and HDI in order to extrapolate their emissions. This is an ergodic assumption, i.e. that the process over time and over the statistical ensemble is the same. Third, we let countries with incomplete data records evolve similarly as their close neighbors (in the emissions-HDI plane, see Fig. 1 in the main text) with complete time series of CO_2 per capita emissions and HDI. Country–based emissions estimates are obtained by multiplying extrapolated CO_2 per capita values by population numbers of three scenarios extracted from the Millennium Ecosystem Assessment report [3].

Finally, we propose a reduction scheme, where countries with an HDI above the development threshold reduce their per capita CO_2 emissions with a rate that is proportional to their HDI. We estimate the minimum proportionality constant so that the global emissions by 2050 meet the 1000 Gt limit.

II. DATA

The analyzed data consists of Human Development Index (HDI), CO_2 emissions per capita values, and Population numbers. In all cases the aggregation level is country scale. Both the HDI and the CO_2 data is incomplete, i.e. the values of some countries or years are missing. In addition, the set of countries with HDI or CO_2 data does not match 100% with the set of countries with population

*Electronic address: luis.costa@pik-potsdam.de
†Electronic address: ca-dr@rybski.de
‡Electronic address: kropp@pik-potsdam.de

data (see Sec. III E).

A. Human Development Index (HDI)

The Human Development Index is provided by the United Nation Human Development Report 2009 and covers the period 1980 to 2007 (in steps of 5 years until 2005, plus the years 2006 and 2007). The data is available for download [4] and is documented [5].

The HDI is intended to reflect three dimensions of human development: (i) a long and healthy life, (ii) knowledge, and (iii) a decent standard of living. In order to capture the dimensions, four indicators are used: life expectancy at birth for "a long and healthy life", adult literacy rate and gross enrollment ratio (GER) for "knowledge", and GDP per capita (PPP US$) for "a decent standard of living". Each index is weighted with 1/3 whereas the "adult literacy index" contributes 2/3 to the education index (knowledge) and gross enrollment index 1/3:

$$\begin{aligned} d_{i,t} &= \frac{1}{3}\left(\frac{LE_{i,t}-25}{85-25}\right) + \\ &\quad \frac{1}{3}\left(\frac{2}{3}\frac{AL_{i,t}}{100}+\frac{1}{3}\frac{GE_{i,t}}{100}\right) + \\ &\quad \frac{1}{3}\left(\frac{\log GDP_{i,t}-\log 100}{\log 40000-\log 100}\right) \quad (1) \\ &= \frac{1}{3}d_{i,t}^{\text{life expectancy}}+\frac{1}{3}d_{i,t}^{\text{education}}+\frac{1}{3}d_{i,t}^{\text{GDP}} \quad , \quad (2) \end{aligned}$$

where $LE_{i,t}$ is the life expectancy, $AL_{i,t}$ the adult literacy, $GE_{i,t}$ the gross enrollment, and $GDP_{i,t}$ the GDP per capita (PPP US$) [6], $d_{i,t}^{\text{life expectancy}}$, $d_{i,t}^{\text{education}}$, and $d_{i,t}^{\text{GDP}}$ denote the corresponding indices. An illustrative diagram can be found in [6]. The components are studied individually in Sec. III B 1.

B. CO_2 emissions per capita

The data on CO_2 emissions per capita is provided by the World Resources Institute (WRI) 2009 and covers the years 1960-2006. The CO_2 emissions per capita are given in units of tons per year. It is available for download [7] and is documented [8].

Carbon dioxide (CO_2) is transformed and released during combustion of solid, liquid, and gaseous fuels [9]. In addition, CO_2 is emitted as cement is calcined to produce calcium oxide. The data does include emissions from cement production but estimates of gas flaring are included only from 1980 to the present. The CO_2 emission values do not include emissions from land use change or emissions from bunker fuels used in international transportation [9].

C. Population

Population projections are provided by the Millennium Ecosystem Assessment Report 2001 and cover the period 2000 to 2100 in steps of 5 years (we only make use of the data until 2050). The data is available for download [10] and is documented [11]. We use the scenarios Adaptive Mosaic (AM), TechnoGarden (TG), and Global Orchestration (GO). We found minimal differences in our results using the Order from Strength (OS) scenario and therefore disregard it. In short:

- The Adapting Mosaic scenario is based on a fragmented world resulting from discredited global institutions. It involves the rise of local ecosystem management strategies and the strengthening of local institutions [11].
- The TechnoGarden scenario is based on a globally connected world relying strongly on technology as well as on highly managed and often-engineered ecosystems to provide needed goods and services.
- The Global Orchestration scenario is based on a worldwide connected society in which global markets are well developed. Supra-national institutions are well established to deal with global environmental problems.

D. Notation

For a country i at year t we use the following quantities:

- Human Development Index (HDI): $d_{i,t}$
- CO_2 emissions per capita: $e_{i,t}^{(c)}$ in tons/(capita year)
- CO_2 emissions: $e_{i,t}$ in tons/year
- cumulative CO_2 emissions: $E_{i,t}$ in tons
- population: $p_{i,t}$

III. EXTRAPOLATING CO_2 EMISSIONS

In this section we detail which empirical findings and assumptions are used to extrapolate per capita emissions of CO_2 and HDI values in a Development As Usual (DAU) approach. The projections are performed statistically, i.e. extrapolating regressions. Our approach is based on 3 assumptions:

1. The Human Development Index, $d_{i,t}$, of all countries evolves in time following logistic regressions (Sec. III A).

2. The Human Development Index and the logarithm of the CO_2 emissions per capita, $e^{(c)}_{i,t}$, are linearly correlated (Sec. III B).

3. The changes of $d_{i,t}$ and $e^{(c)}_{i,t}$ are correlated among the countries, i.e. countries with similar values comprise similar changes (Sec. III C).

By Development As Usual we mean that the countries behave as in the past, with respect to these 3 points. In particular, past behavior may be extrapolated to the future.

It is impossible to predict how countries will develop and how much CO_2 will be emitted per capita. Accordingly, we are not claiming that the calculated extrapolations are predictions. We rather present a plausible approach which is supported by the development and the emissions per capita in the past. We provide the estimates consisting of projected HDI and emission values as supplementary material.

A. Extrapolating Human Development Index (HDI)

We elaborate the evolution of HDI values following a logistic regression [12]. This choice is supported by the fact that the HDI is bounded to $0 \leq d_{i,t} \leq 1$ and that the high HDI countries develop slowly. Therefore, we fit for each country separately

$$\tilde{d}_{i,t} = \frac{1}{1 + \mathrm{e}^{-a_i t + b_i}} \tag{3}$$

to the available data (obtaining the parameters a_i and b_i), whereas we only take into account those countries for which we have at least 4 measurement points, which leads to regressions for 147 countries out of 173 in our data set. Basically, a_i quantifies how fast a country develops and b_i represents when the development takes place. Figure 2 in the main paper depicts a collapse (see e.g. [13]) of the past HDI as obtained from the logistic regression. It illustrates how the countries have been developing in the scope of this approach.

Based on the obtained parameters, a_i and b_i, we estimate the future HDI of each country assuming similar development trajectories as in the past. Table S1 lists those countries which pass $d_{i,t} = 0.8$ [5] until 2051 and provides periods when this is expected to happen according to our projections. Further, we expect from the extrapolations that before 2021 more people will be living in countries with HDI above 0.8 (see main text) than below. In addition, until 2051 around 85% will be living in countries with HDI above 0.8.

The logistic regression, Eq. (3), is in physics also known as Fermi-Dirac distribution. It comprises three distinct points. The inflection point is located at $t = 0$ and $d = 0.5$ for $a_i = 1$ and $b_i = 0$. Two other distinct points are those of maximum or minimum curvature. They are located at $t = -\ln(2 \pm \sqrt{3})$ and $d = (3 \pm \sqrt{3})^{-1}$, i.e. $d \approx 0.21 \vee d \approx 0.79$. Accordingly, from a geometrical point of view, $d^* = 0.8$ is a reasonable threshold. The approach of fitting logistic regressions to country data is also been used in other fields, see e.g. [14].

B. Estimating CO_2 emissions per capita

In Figure 1 of the main text we find among the ensemble of countries correlations between the HDI, $d_{i,t}$, and the CO_2 emissions per capita, $e^{(c)}_{i,t}$ (see also Fig. S4). We apply the exponential regression

$$\hat{e}^{(c)}_{i,t} = \mathrm{e}^{h_t d_{i,t} + g_t} \tag{4}$$

to the country data by linear regression [15] through $\ln e^{(c)}_{i,t}$ versus $d_{i,t}$ for fixed years t and obtain the parameters h_t and g_t as displayed in the panels (c) and (d) of Fig. 3 in the main text, respectively.

We take advantage of these correlations and assume that the system is ergodic, i.e. that the process over time and over the statistical ensemble are the same. In other words, we assume that these correlations [main text Fig. 1, Eq. (4)] also hold for each country individually, and apply the exponential regression:

$$\tilde{e}^{(c)}_{i,t} = \mathrm{e}^{h_i d_{i,t} + g_i} \quad . \tag{5}$$

Thus, for each country, we obtain the parameters h_i and g_i, characterizing how its emissions per capita are related to its development (or vice versa). Note that while in Eq. (4) the year t is fixed, leading to the time-dependent parameters h_t and g_t, in Eq. (5) the country i is fixed, leading to the country-dependent parameters h_i and g_i. This regression, Eq. (5), is applied to 121 countries for which sufficient data is available, i.e. at least 4 pairs $e^{(c)}_{i,t}$ and $d_{i,t}$. Based on extrapolated HDI values we then calculate the corresponding future emissions per capita estimates. Figure S1 shows for 9 examples the past and extrapolated values of emissions per capita.

1. *Correlations between CO_2 emissions per capita and HDI components*

In addition to the correlations between CO_2 emissions per capita and the HDI, we also calculated the correlations between CO_2 emissions per capita and the three components of the HDI, i.e. a long and healthy life, knowledge, and a decent standard of living (see Sec. II A). As can be seen in Fig. S2 for the year 2006, in all cases we find clear correlations. In particular, we find that the slopes for the components are smaller than the one for HDI, see Tab. S2. This supports the usage of the HDI as summary measure. However, the correlation coefficients of the life expectancy index vs. CO_2 emissions per capita and the education index vs. CO_2 emissions per capita are

somewhat smaller (0.78 and 0.82, respectively) than the one for the GDP index vs. CO_2 emissions per capita (0.92).

By plotting the evolution of individual HDI components one can e.g. see that relative gains in education and life expectancy in Bangladesh supplant the gains in per capita GDP (Fig. S2). Obviously, the components them self are also correlated among each other (not shown).

C. Estimating values for missing countries

For 52 countries out of 173 the available data is not sufficient, i.e. there are not enough values to perform the regressions Eq. (3) or Eq. (5). In order not to disregard these countries we take advantage of correlations, i.e. countries with similar HDI have on average similar changes of HDI as well as countries with similar emissions per capita have on average similar changes of emissions. In other words, in the $\ln e^{(c)}_{i,t}$-$d_{i,t}$-plane, the countries move similarly to their neighborhood.

Figure 3(c) and (d) in the main text also shows how the regressions to the emissions per capita versus the HDI evolve. The slope, h_t, becomes larger and the intercept, g_t, smaller. In both cases the standard error remains approximately the same, showing that the spreading of the cloud does not change. In other words, if the countries would develop independently from each other, then the error bars should increase with time.

In order to further support this feature, in Fig. S3 we show the correlations for both quantities. Thus, for each pair of countries i and j (that are in the set with sufficient data), we calculate

$$c_{i,j}(\Delta d) = \frac{(\delta d_i - \langle \delta d \rangle)(\delta d_j - \langle \delta d \rangle)}{\sigma^2_{\delta d}} \quad , \tag{6}$$

where $\delta d_i = d_{i,2005} - d_{i,2000}$ is the difference in time, $\langle \delta d \rangle$ is the average of δd among all countries providing enough data, and $\sigma^2_{\delta d}$ is the corresponding variance. In Fig. S3(a) $c_{i,j}$ is plotted against $\Delta d_{i,j} = |d_{j,2000} - d_{i,2000}|$, the difference in space of the considered pair of countries. One can see that the correlations decay exponentially following $\tilde{c}_{i,j}(\Delta d) \approx \mathrm{e}^{-67.8 \Delta d - 0.66}$. This indicates that countries that have similar HDI also develop similarly.

For the emissions per capita we perform the analogous analysis, replacing δd_i by $\delta \ln e^{(c)}_i$ in Eq. (6) and consequently in the quantities $\langle \ln \delta e^{(c)} \rangle$, $\sigma^2_{\delta \ln e^{(c)}}$, and $\Delta \ln e^{(c)}_{i,j}$. In Fig. S3(b) we obtain similar results as for the HDI. For the emissions, the correlations decay as $\tilde{c}_{i,j}(\Delta \ln e^{(c)}) \approx \mathrm{e}^{-1.82 \Delta \ln e^{(c)} - 2.27}$. For both, δd_i and $\delta \ln e^{(c)}$, the correlations were calculated between the years 2000 and 2005.

We take advantage of these correlations and utilize them to extrapolate $d_{i,t}$ and $e^{(c)}_{i,t}$ by using the estimated correlation functions as weights. The change in development of a country k, belonging to the set of countries without sufficient data, we calculate with

$$\delta d_k = \frac{\sum_j (\tilde{c}_{k,j}(\Delta d) \delta d_j)}{\sum_j \tilde{c}_{k,j}(\Delta d)} \quad , \tag{7}$$

where the index j runs over the set of countries with sufficient data. Then, the HDI in the following time step is

$$d_{k,t+1} = d_{k,t} + \delta d_{k,t} \quad . \tag{8}$$

The analogous procedure is performed for the emissions per capita.

The results are shown in Fig. S4. For comparison, the panels (a) and (b) show the measured values for the years 2000 and 2006. The panels (c) to (g) exhibit the extrapolated values, whereas the black dots belong to the set of countries with sufficient data (only HDI-extrapolation and HDI-CO_2-correlations) and the brownish dots belong to the set of countries without sufficient data. In sum we can extrapolate the emissions for 172 countries (for one there is no 2006 emissions value). For most countries we obtain reasonable estimations (see also Sec. III E). Panels (h) and (g) show the corresponding parameters h_t and g_t (slope and intercept). The extrapolated values follow the tendency of the values for the past, supporting the plausibility of this approach. Nevertheless, the standard errors increase slightly in time, which indicates that the cloud of dots becomes slightly more disperse, i.e. weaker ensemble correlations between $e^{(c)}_{i,t}$ and $d_{i,t}$.

Figure S5 summarizes how the regressions – Eq. (4) to the cloud of points representing the countries – evolve in the past and according to our projections. Since the countries develop, the regression line moves towards larger values of the HDI and at the same time its slope becomes steeper. As a consequence, on average the per capita emissions of countries with $d_{i,t} \simeq 0.8$ decrease with time from approx. 5.2 tons per year in 1980 to approx. 2.9 tons per year in 2005 and we expect it to reach approx. 1.5 tons per year in 2051. This is in line with previous analysis [16].

D. Uncertainty

In order to obtain an uncertainty estimate of our projections, we take into account the residuals of the regressions to the HDI versus time and CO_2 versus HDI. Thus, we calculate the root mean square deviations, $\sigma^{(d)}_i$ and $\sigma^{(e)}_i$, respectively. The upper and lower estimates of emissions per capita are then obtained from

$$\tilde{d}_{i,t,(\pm)} = \frac{1}{1 + \mathrm{e}^{-a_i t + b_i \mp \sigma^{(d)}_i}} \tag{9}$$

and

$$\tilde{e}^{(c)}_{i,t,(\pm)} = g_i \mathrm{e}^{h_i \tilde{d}_{i,t,(\pm)} \pm \sigma^{(e)}_i} \quad . \tag{10}$$

In a rough approximation, assuming independence of the deviations, the upper and lower bounds correspond to the range enclosing 90%.

The obtained ranges can be seen as gray bands in Fig. S1 and S6. We find that the global cumulative CO_2 emissions between the years 2000 and 2050 discussed in the main text exhibit an uncertainty of approx. 12% compared to the typical value, which also includes uncertainty due to the population scenarios (see Sec. II C and Fig. 4 in the main text).

The global emissions we calculate for the years 2000 and 2005 (i.e. multiplying recorded CO_2 emissions per capita with recorded population numbers, see Sec. IV) are by less than 2% smaller than those provided by the WRI [7]. This difference, which can be understood as a systematic error, can have two origins. (i) Some countries are still missing. Either they are not included in the data at all, or they cannot be considered, such as when we multiply emissions per capita with the corresponding population and the two sets of countries do not match. (ii) The population numbers we use might differ from the ones WRI uses.

E. Limitations

Since countries with already large HDI can only have small changes in $d_{i,t}$, the emissions per capita also do not change much. For example, for Australia, Canada, Japan, and the USA we obtain rather stable extrapolations (Fig. S1 and S6). This could be explained by the large economies and the inertia they comprise. In contrast, for some countries with comparably small populations, the extrapolated values of emissions per capita reach unreasonably high values, such as for Qatar or Trinidad&Tobago in Fig. S6.

Since one may argue that countries with large populations should have more weight [16] when fitting the per capita emissions versus the HDI, Eq. (4), in Fig. S7, for the year 2000, we employ three ways of weighing. While the solid line is the fit where all countries have the same weight, the dotted line is a regression where the points are weighted with the logarithm of the country's population. We found that it is almost identical to the unweighted one. In contrast, the dashed line is a regression where the points are weighted with the population of the countries (not their logarithm as before). The obtained regression differs from the other ones and as expected it is dominated by the largest countries (five of them are indicated in Fig. S7). However, this difference does not influence our extrapolations since we do not use the ensemble fit, Eq. (4), but instead regressions for individual countries, Eq. (5).

F. Enhanced development approach

In addition to the DAU approach, we also tested one of enhanced development where we force the countries with $d < 0.8$ to reach an HDI of 0.8 by 2051. This can be done by parameterizing the HDI-regression through two points, namely $d_{i,2006}$ and $d_{i,2050} = 0.8$, instead of fitting Eq. (3). The corresponding emission values can then be estimated by following the ensemble fit, Eq. (4). Nevertheless, since the relevant countries are rather small in population and still remain with comparably small per capita emissions, the difference in global emissions is minor, namely at most an additional 3% (cumulative emissions until 2050, GO population scenario). Thus, we do not further consider this enhanced development.

IV. CUMULATIVE EMISSIONS

To obtain the cumulative emission values, shown in Fig. 2 of the main text, we perform the following steps:

1. Estimate the emissions per capita, $e^{(c)}_{i,t}$, according to the descriptions in Sec. III.
2. Multiply the per capita emissions with the population of the corresponding countries, $e_{i,t} = e^{(c)}_{i,t}\, p_{i,t}$, resulting in the total annual emissions of each country.
3. Calculate the cumulative emissions by integrating the annual emission values, $E_{i,t} = \sum_{\tau=t_0}^{t} e_{i,\tau}$, where we choose $t_0 = 2000$.

The intersection of the set of countries with projected per capita emission values with the set of countries with projected population values consists of 165 countries.

V. REDUCTION SCHEME

In the main text we propose a CO_2-reduction scheme which is in line with our results. The reduction rate of the individual countries should depend on their individual HDI value. Thus, a country i reduces it's per capita emissions at year t according to

$$e^{(c)}_{i,t-5y} \;\rightarrow\; (1 - r_{i,t})\, e^{(c)}_{i,t} \tag{11}$$

with the 5-year reduction rate, $r_{i,t}$, which depends on the country's HDI following

$$r_{i,t} = f\,(d_{i,t} - d^*) \quad \text{for} \quad d_{i,t} > d^* \;, \tag{12}$$

involving two parameters, the development threshold, $0 < d^* < 1$, and the proportionality constant, $f > 0$. The former determines at which HDI the countries start their reduction of per capita CO_2 emissions and the latter determines how strong the reduction rate increases with increasing HDI.

Obviously, the larger d^* is, the larger f needs to be (and vice verse) so that global emissions can be limited. Choosing the development threshold, $d^* = 0.8$, we estimate that $f \simeq 3.3$ would lead to global cumulative emissions ranging between 850 and 1100 Gt of CO_2 by 2050 if reduction starts in 2015 (assuming the same uncertainty as in DAU).

Naturally, larger values of f lead to smaller global emissions ($f \simeq 3.3$ is a lower bound). However, the response is non-linear: $d^* = 0.7$ requires $f \simeq 1.1$ and $d^* = 0.6$ only $f \simeq 0.6$. For $d^* > 0.8$ the emissions can practically not be restricted to the limit of 1000 Gt global emissions by 2050 within the proposed reduction framework.

Acknowledgments

We thank M. Boettle, S. Havlin, A. Holsten, N. Kozhevnikova, D. Reusser, H.D. Rozenfeld, J. Sehring, and J. Werg for discussions and comments. Furthermore, we thank A. Schlums for help with the manuscript. We also acknowledge the financial support from EU funded projects ENSURE (2008-2011) and BaltCICA (Baltic Sea Region Programme 2007-2013).

[1] S. Chakravarty, A. Chikkatur, H. de Coninck, S. Pacala, R. Socolow, and M. Tavoni, Proc. Nat. Acad. Sci. U.S.A. **106**, 11884 (2010).
[2] J. M. Rhemtulla, D. J. Mladenoff, and M. K. Clayton, Proc. Nat. Acad. Sci. U.S.A. **106**, 6082 (2008).
[3] J. Alcamo, J. Alder, E. Bennett, E. R. Carr, D. Deane, G. C. Nelson, and T. Ribeiro, *Millennium Ecosystem Assessment* (Island Press, 2005).
[4] United Nation Human Development Report (2009), http://hdr.undp.org/en/reports/global/hdr2009/ .
[5] UNDP, *Human development report 2009: Overcoming barriers: Human mobility and development* (2009), http://hdr.undp.org/en/media/HDR_2009_EN_Indicators.pdf .
[6] UNDP, Tech. Rep., United Nations (2008).
[7] World Resources Institute (2009), http://earthtrends.wri.org/searchable_db/results.php?years=all&variable_ID=466&theme=3&country_ID=all&country_classification_ID=all .
[8] World Resources Institute (2009), http://earthtrends.wri.org/searchable_db/variablenotes.php?varid=466&theme=3 .
[9] World Resources Institute, Climate Analysis Indicators Tool (2009), http://cait.wri.org .
[10] Millennium Ecosystem Assessment Report (2001), http://wdc.nbii.gov/ma/datapage.htm .
[11] Millennium Ecosystem Assessment Report (2001), http://www.millenniumassessment.org/documents/document.332.aspx.pdf .
[12] D. W. Hosmer and S. Lemeshow, *Applied Logistic Regression*, Wiley Series in Probability and Statistics (John Wiley & Sons, New York, 2000), ISBN 0-471-35632-8.
[13] R. D. Malmgren, D. B. Stouffer, A. S. L. O. Campanharo, and L. A. N. Amaral, Science **325**, 1696 (2009).
[14] L. Hu, K. Tian, X. Wang, and J. Zhang, online-arXiv **arXiv:1105.5891v1 [q-fin.GN]** (2011), URL `http://arxiv.org/abs/1105.5891`.
[15] R. L. Mason, R. F. Gunst, and J. L. Hess, *Statistical Design and Analysis of Experiments – with applications to engineering and science* (John Wiley & Sons, New York, 1989), ISBN 0-471-85364-X.
[16] J. K. Steinberger and J. T. Roberts, Ecol. Econ. **70**, 425 (2010).

FIG. S1: Examples of extrapolated CO_2 emissions per capita. For the countries with top total emissions in 2000, we plot the measured values (solid lines) and extrapolated values up to the middle of the 21st century (dotted lines). The gray uncertainty range is obtained by including the statistical errors of the regressions (one standard deviation each).

FIG. S2: Correlations between CO_2 emissions per capita and HDI as well as its components. Panels (a-d) are cross-plots in semi-logarithmic representation, where each filled circle represents a country. (a) depicts the CO_2 emissions per capita values versus the corresponding HDI values for the year 2006 (172 countries). (b-d) depict the analogous for the HDI components, i.e. (b) GDP index, (c) life expectancy index, and (d) education index. The slopes and correlation coefficients are listed in Tab. S2. The Panels also include the trajectories (1980-2006) of Japan (green diamonds), China (blue triangle up), India (grey triangle right), and Bangladesh (cyan $\times$) evolving from the lower left to the upper right. The solid straight lines are exponential fits, Eq. (4), to the data and the dotted lines in (b-d) correspond to the fit from (a).

FIG. S3: Correlations of the changes in development and emissions per capita. For observed data between the years 2000 and 2005, we plot in (a) the correlation function, Eq. (6), of the temporal changes of the HDI as a function of the difference of the countries in terms of HDI. In (b) the analogue, namely the correlation function of the temporal changes of the emissions per capita is plotted as a function of the difference in terms of emissions per capita. While the green dots represent the products of individual pairs, the blue filled circles represent the averages in logarithmic bins.

FIG. S4: Correlations between CO_2 emissions per capita and HDI. Panels (a) to (g) are cross-plots in semi-logarithmic representation, where each filled circle represents a country, for past years (a) 2000: 135 countries and (b) 2006: 172 countries, as well as extrapolated (c) to (g) 2011-2051 (172 countries each). The brownish circles represent those countries, which due to missing data have been estimated assuming correlations in the changes of $d_{i,t}$ as well as $e_{i,t}^{(c)}$ (see Sec. III C). Panels (h) and (i) show how the parameters h_t and g_t evolve in time (the open symbols are obtained from the extrapolated values of all countries). Both parameters are based on only those 71 countries providing HDI and CO_2 values for all years 1980, 1985, 1990, 1995, 2000, 2005, 2006. The qualitative agreement of h_t and g_t between past and extrapolated supports the plausibility of the presented approach. The error bars are given by the standard errors. The panels (h) and (i) are the same as Fig. 3(c) and (d) in the main text.

FIG. S5: Evolving correlations between CO_2 emissions per capita and HDI. The lines represent the linear regressions applied to the logarithm of CO_2 emissions per capita versus HDI for the past (solid lines) and our projections (dashed lines). The numbers at the right edge correspond to the $e_{i,t}$ for which the regressions cross $d_{i,t} = 0.8$ in 1980, 2005 and projected for 2051.

FIG. S6: Examples of extrapolated CO_2 emissions per capita. For the countries with top emissions per capita in 2006, we plot the measured values (solid lines) and extrapolated values up to the middle of the 21st century (dotted lines). Qatar and Luxembourg belong to those countries, which due to missing data have been extrapolated utilizing correlations in the changes of $d_{i,t}$ as well as $e_{i,t}^{(c)}$ (Sec. III C). The gray uncertainty range is obtained by including the statistical errors of the regressions (one standard deviation each). Analogous to Fig. S1 but for different countries.

FIG. S7: Correlations between CO_2 emissions per capita and HDI. For the year 2000 three different ways of performing a regressions are exemplified. Solid line in the background: the regression when each country has the same weight. Dotted line: the countries have weights according to the logarithm of their population. Dashed line: the countries have weights according to their population. For comparison the five most populous countries are highlighted.

TABLE S1: Periods during which countries are expected to pass the HDI value of 0.8 according to the extrapolations. The rows denote the countries and the columns denote periods of five years. The transitions are indicated with •.

TABLE S2: Slopes and correlation coefficients of the exponential fits, Eq. (4), applied to the HDI and it's components.
