# Supplementary material for: A Human Development Framework for CO2 Reductions
Source: PLoS One. 2011 Dec 21;6(12):e29262. doi: 10.1371/journal.pone.0029262 (PMC3244443; doi:10.1371/journal.pone.0029262)
Supplement: Table S1 — Periods during which countries are expected to pass the HDI value of 0.8 according to the extrapolations. The rows denote the countries and the columns denote periods of five years. The transitions are indicated with •. (PDF) [file pone.0029262.s008.pdf]

	2007 2011	2012 2016	2017 2021	2022 2026	2027 2031	2032 2036	2037 2041	2042 2046	2047 2051
Armenia	•								
Colombia	•								
Iran	•								
Kazakhstan	•								
Mauritius	•								
Peru	•								
Turkey	•								
Ukraine	•								
Azerbaijan		•							
Belize		•							
China		•							
Dominican.Republic		•							
El.Salvador		•							
Georgia		•							
Jamaica		•							
Maldives		•							
Samoa		•							
Suriname		•							
Thailand		•							
Tonga		•							
Tunisia		•							
Algeria			•						
Bolivia			•						
Fiji			•						
Honduras			•						
Indonesia			•						
Jordan			•						
Sri.Lanka			•						
Syrian.Arab.Republic			•						
Turkmenistan			•						
Viet.Nam			•						
Cape.Verde				•					
Egypt				•					
Equatorial.Guinea				•					
Guatemala				•					
Guyana				•					
Mongolia				•					
Paraguay				•					
Philippines				•					
Kyrgyzstan					•				
Nicaragua					•				
Uzbekistan					•				
Lao.People's.Democratic.Republic						•			
Morocco						•			
Vanuatu						•			
Botswana							•		
India							•		
Nepal							•		
Bangladesh								•	
Sao.Tome.and.Principe								•	
Yemen								•	
Bhutan									•
Ethiopia									•
Pakistan									•
Solomon.Islands									•
Uganda									•
